# Role of surgery in advanced/metastatic renal cell carcinoma

**DOI:** 10.4103/0970-1591.65381

**Published:** 2010

**Authors:** Suresh Bhat

**Affiliations:** Department of Urology, Medical College, Kottayam-686 008, Kerala, India

**Keywords:** Cytoreductive nephrectomy, immunotherapy, metastasectomy, metastatic renal cancer, targeted molecular therapy

## Abstract

Metastatic renal cell cancer (RCC) is a malignant disease without curative treatment. These patients are usually symptomatic and desperate for effective palliative treatment. Radiotherapy, chemotherapy, and hormonal therapy are not effective in these patients. A multimodal approach consisting of cytoreductive nephrectomy, systemic therapy (which includes cytokines or targeted molecules), and metastasectomy have been shown to be useful in prolonging the survival and improving the quality of life in a select group of patients with metastatic renal cancer. Patients with oligometastatic disease, good performance status, and delayed presentation of the secondaries have better results following this integrated approach. Although there is some controversy regarding the order in which nephrectomy and systemic therapy are to be instituted, well-controlled studies like the South West Oncology Group and European organization research and treatment of cancer have shown that upfront nephrectomy gives better survival compared to neoadjuvant systemic therapy followed by nephrectomy. This order is the standard presently. Of late, with better understanding of the genetic basis and the biology of the various subtypes of renal cell carcinoma, targeted molecular therapies have emerged as an equally effective alternative therapy to cytokines. Recent reports have proven that targeted therapy is more effective with comparable side effects. Metastasectomy in a subgroup of patients improves survival and quality of life specifically in those with lung secondaries and painful bone metastases.

## INTRODUCTION

Renal cell carcinoma (RCC) is a serious and lifethreatening disease. It accounts for about 2% of all cancers with a worldwide annual increase of 1.5-5.5%. This is mainly due to enhanced detection of tumors by increased use of imaging techniques.[1] RCC is the most lethal among the urinary tract tumors. It has a highly variable natural history and biological behavior. Approximately 30-40% patients with malignant renal cortical tumors will either present with or later develop metastatic disease.[2] After radical or partial nephrectomy, metastases develop in about 2 years.[3] About 90% of the metastases are from the conventional (clear cell) RCC.[3] The 5 year survival for all stages of RCC has continued to improve. Disease-free interval (DFI) of up to 30 years has been reported. Patients with untreated metastatic disease have a 5 year survival of 0-18%.[4]

Kidney cancer is not a single disease; it is made up of a number of different types of cancers, each with a different histology, different clinical course, and caused by alteration of different genes. Metastases have been reported to almost all the organs in the body. The common sites include lungs, liver, bones, adrenals, pancreas, brain, thyroid, skin, and ureter. Conventional RCC metastasizes commonly to the lungs, whereas papillary and chromophobe variety to the lymph nodes and the liver, respectively.[4] Median patient survival for patients with metastasis is about 10-12 months.[5] Patients with metastatic disease have an 18% chance of surviving 2 years.[6] However, subsets of patients with advanced disease have shown improved survival. The following factors predict the risk of metastasis following radical nephrectomy (RN) for clinically localized RCC viz: size and stage of primary tumor, extent of regional lymph node involvement, if any, tumor histology, presence or absence of necrosis, and presence or absence of vascular invasion.

A decade ago, patients with metastatic RCC (mRCC) had very dismal prognosis. Now, the outlook has changed remarkably thanks to the tremendous advancements in the field of medical management of mRCC especially immunotherapy (IT), targeted therapy, and the better understanding of the role and timing of cytoreductive nephrectomy (CRN) as well as expertise in minimally invasive surgery. Surgical intervention in any patient with mRCC has one of the two aims viz: (1) complete metastasectomy to render the patient clinically free of all sites of metastases or (2) cytoreductive nephrectomy to remove the primary tumor either before or after the initiation of systemic therapy. Whether to proceed with metastasectomy depends on multiple factors like the sites and number of metastases, resectability, surgical expertise, and patient compliance and general condition. The decision to perform metastasectomy is often empirical. Surgery in the setting of mRCC may be in the form of palliative nephrectomy, nephrectomy as a component of adoptive immunotherapy, cytoreductive nephrectomy, and metastasectomy.

## SURGICAL CONSIDERATIONS IN PATIENTS WITH mRCC

Radical nephrectomy in the setting of mRCC, commonly called cytoreductive nephrectomy, is usually advocated as part of multimodality treatment approach. Over the years, several arguments have been put forward to support the concept of radical nephrectomy for mRCC. It is common knowledge that nephrectomy in a patient with mRCC almost certainly cannot bring cure and that these patients die of their metastases than the primary tumor. In 1978, deKernion and colleagues showed that nephrectomy alone had a minimal effect on survival in mRCC patients.[7] In fact, the only rationale could be, it may bring about a survival benefit and improvement in quality of life (QOL). However, with the advent of modern targeted molecular therapy (TMT), a critical reevaluation of the approach to the management of patients with mRCC is becoming increasingly significant.

### The role of palliative nephrectomy

In general, palliative nephrectomy alone for metastatic disease without adjuvant therapy is not useful. As the general condition of the patient is poor most of the time, surgery may be associated with morbidity and mortality. It should only rarely be done in patients with intractable pain, bleeding, uncontrolled hypertension, symptoms due to paraneoplastic syndromes such as uncontrolled hypercalcemia, erythrocytosis, if usual measures fail. However, the systemic effects attributed to RCC may be produced by the metastases and not necessarily due to the primary tumor itself. Hence, palliative nephrectomy may not bring relief for the problem which it was intended to palliate. Walther and colleagues in 12 patients with mRCC and hypercalcemia found that after nephrectomy, calcium decreased in only 7 patients, it increased in 4 patients and remained unchanged in 1 patient.[8] In addition, patients who had reduction in serum calcium did not fare better than those who did not show any reduction in the calcium, in that both groups had median survival of 6 months. As minimally invasive procedures like angioinfarction of the tumor result in equally effective palliation, the role of palliative nephrectomy may be limited. However, the quality of life in selected patients after palliative nephrectomy appears better.

### Nephrectomy as a component of adoptive immunotherapy

Adoptive immunotherapy involves transfer of antitumor cells into the host to mediate tumor regression. Nephrectomy is required to harvest tumor antigens or tumor infiltrating lymphocytes (TILs). University of California at Los Angeles (UCLA) has reported some encouraging results with this modality.[9] In 55 patients, an overall partial response (PR) rate of 25.5% and complete response (CR) of 9.1% was noted. There was a survival advantage of 15 months in those patients receiving tumor infiltrating lymphocytes and interleukin 2 (IL-2). In a multicenter trial, combination of tumor infiltrating lymphocytes with low dose IL-2 was compared with IL-2 alone. Treatment with CD8+ tumor infiltrating lymphocytes did not improve response rate or survival in patients treated with IL-2 post-nephrectomy.[10] Although the results of adoptive immunotherapy are disappointing, with proper informed consent, more patients need to be enrolled into clinical trials. Future trials are needed to document the efficacy of adoptive immunotherapy.

## CYTOREDUCTIVE NEPHRECTOMY

### Biologic rationale for cytoreductive nephrectomy

RCC is an immunogenic tumor as evidenced by the expression of multiple tumor antigens notably CA IX (carbonic anhydrase). RCC has the ability to manipulate and suppress the host’s natural immunity leading to immunological dysfunction. The primary tumor might suppress the antitumor effect of the host defense mechanism. It suppresses the cell-mediated immunity. The primary tumor acts as an ‘immunogenic sink’ whereby it diverts the circulating macrophages, lymphocytes, and immunoglobulins away from the distant metastases.[11] Lymphocytes from patients with mRCC have been shown to have defective T-cell receptors, enhanced apoptosis, and defective signal transduction with tumor infiltrating lymphocytes showing greater dysfunction than peripheral lymphocytes.[12] RCC produces high levels of proinflammatory and T-cell inhibitory substances such as IL-8, IL-6, IL-10,TNF, and TGFb-1 all of which suppress immunologic responses.[12] The primary tumor also lacks response to immunotherapy. Hence, removal of this large load of immunosuppressive tumor may improve the host’s immune surveillance. A reduction in the tumor burden increases the likelihood of response. Additional immunotherapy can augment the host immune mechanisms thereby producing better survival and quality of life.

This is typified by the occasional and rare phenomenon of spontaneous disappearance of the metastases, especially in the lungs. The lungs are rich in macrophages, lymphocytes, and immunoglobulins. This spontaneous regression is due to host-mediated cytotoxicity. This occurs in 0.4-0.8% of patients. In the national cancer institute (NCI) series of 91 patients, 4 patients showed complete regression of metastases after nephrectomy.[13] However, most of these lesions were not biopsy proven and could have been old granulomas, fungal lesions, or pulmonary infarcts. Spontaneous regression is a rare event and nephrectomy should not be done for this purpose alone. Another aim of nephrectomy in mRCC is to improve the quality of life by obtaining relief of symptoms like hematuria, pain and systemic symptoms. Mostly, however, the pain is due to involvement of the nerves and the bones, and surgical treatment is usually inadequate. Some patients may have psychological benefit with a feeling that the cancer has been removed. Another benefit of nephrectomy is removal of source of metastases. This results in resetting of the clock, for accumulation of lethal tumor burden.

There is some controversy regarding the timing of nephrectomy in the multimodal approach. There are authors who prefer upfront nephrectomy and others who perform nephrectomy, only after systemic therapy. Both have their own pros and cons.

### Nephrectomy before systemic therapy

Cytoreductive nephrectomy (debulking nephrectomy) has an important role in the multi-modal management of mRCC. Even though there is a controversy regarding the timing of nephrectomy, most of the authors feel that it is beneficial to perform the cytoreductive nephrectomy before the planned systemic therapy. Proceeding with cytoreductive nephrectomy in patients with good performance status (PS) and easily resectable primary is reasonable and much followed option.

Cytoreductive nephrectomy appears to be beneficial for many patients with mRCC. However, it is not curative and should not be done indiscriminately. Nephrectomy alone offers no benefit, however, when done as part of multimodal treatment approach, it does have a complementary role. When evaluating the controversial issue of nephrectomy before immunotherapy or TMT, the following issues need to be looked into. They are (a) after nephrectomy, will the patient be stable enough to receive the systemic therapy and (b) will initial nephrectomy improve the objective response of systemic therapy at metastatic sites. Obviously, patient selection is the most crucial factor in this. Patients with other comorbidities like compromised cardiac and pulmonary function cannot be part of this protocol. Patients who are most likely to benefit from cytoreductive nephrectomy include those with substantial tumor burden, (in excess of 75%) in the affected kidney, good performance status, and no central nervous system or liver metastases.[14] Removing the primary tumor may also prevent further seeding of metastases and eliminate potential source of pain and hemorrhage. Cytoreductive nephrectomy leaves behind a smaller volume of cancer cells which are easier to be managed with systemic therapy. These form the basis of performing the nephrectomy first. Patients with normalization of the C-reactive protein after nephrectomy have a better survival.[15] Factors that may militate against nephrectomy include comorbidities that increase the risk of surgery and high volume of metastatic disease. Kader *et al* maintain that it is the physiological age and not the chronological age that should be considered before taking up patients for cytoreductive nephrectomy.[16]

Potential disadvantages of cytoreductive nephrectomy are perioperative morbidity and mortality, and delay in starting systemic therapy. Many patients due to the ensuing complications become unfit to receive the systemic therapy and most patients do not respond to immunotherapy.[17] The mortality of cytoreductive nephrectomy varies from 6 to 11% and the morbidity is around 20%.[17] In the South West Oncology Group (SWOG) trial, there was only one death in the perioperative period.[18] Expert surgeons can now perform even challenging resections using laparoscopic techniques. This may reduce the complication rate. Reports by Bennet and associates, National Cancer Institute and Cleveland Clinic showed that a significant number of patients (22-77%) could not receive immunotherapy.[19–21] In the SWOG trial, only 2% patients were unable to receive interferon after nephrectomy.[18]

The best support for the pre-immunotherapy nephrectomy came from two prospective, randomized studies by the SWOG and European organization research and treatment of cancer (EORTC) groups. In the SWOG study, the median survival for the cytoreductive nephrectomy + immunotherapy group was 11.1 months compared to 8.1 months in the interferon (IFN) only group. This represents a 31% reduction in the risk of death (*P*=0.002). Thus, cytoreductive nephrectomy appears to significantly improve overall survival in patients with mRCC treated with IFN-α. This effect was independent of performance status, site of metastases, and the presence of measurable disease. Although the result is statistically significant, the overall survival advantage is only 5.8 months.[18] In the EORTC study, the survival was 17 and 7 months, respectively.[22] Flanigan and colleagues did a combination analysis of these two studies and found a median survival of 13.6 months for the combination group and 7.8 months for the immunotherapy alone patients. There was a survival advantage of about 6 months for the cytoreductive nephrectomy + immunotherapy group.[23] Unlike other series, operative mortality in the combined experience was only 1.5% and only 5.6% of patients did not receive IFN. A provocative study from the SWOG hypothesized that the survival advantage could be due to the post-operative azotemia resulting from cytoreductive nephrectomy and not due to the removal of the tumor.[24] Many tumors acidify their peritumoral microenvironment as a means of overcoming the negative effects of the intracellular acidosis that results from tumor cell hypoxia and increased glycolytic metabolism. Unilateral nephrectomy may alter the dynamics of the tumor host interface and further acidify the tumor pH sufficiently to exceed the tolerance of the tumor cells, slowing or reversing tumor growth and invasion. In this SWOG study, patients developing increase in blood urea nitrogen (BUN) and creatinine had a significantly improved survival compared with those who did not (17 vs 4 months).

According to Bromwich and colleagues, this advantage could be due to referral pattern, surgical judgments, and patient selection.[25] In their group of 20 patients, who underwent cytoreductive nephrectomy, 13 received immunotherapy, yet the median survival was only 9.5 months. Kassouf and associates have shown that cytoreductive nephrectomy followed by systemic therapy is equally good for non-clear histology mRCC also.[26] These patients compared to the clear cell mRCC subtype were younger, had higher lymph node involvement, and sarcomatoid variety. That is why, after metastasis, the non-clear variety has worse prognosis than the clear cell type.

Walther *et al* used laparoscopic techniques for cytoreductive nephrectomy in an effort to reduce the morbidity so that systemic therapy could be initiated earlier.[27] They compared open nephrectomy, lap-assisted nephrectomy, and lap morcellation in relation to starting the immunotherapy. For open surgery patients, it took a median time interval of 67 days (56-151 days), whereas for lap-assisted patients, it was 60 days (47-63 days). The group that benefited the most was those who had morcellation. In these patients, systemic therapy could be started at a median of 37 days (37-57 days). The authors concluded that laparoscopy offered a reasonable method of performing nephrectomy in preparation for immunotherapy.

A Cochrane-based analysis concluded that in fit patients with metastases at diagnosis and minimal symptoms, nephrectomy followed by IFN-α gives the best survival strategy for fully validated therapies.[28] So far, only, cytoreductive nephrectomy followed by immunotherapy is authoritatively evaluated and approved. It constitutes standard therapy currently.[29]

### Nephrectomy after systemic therapy

Many clinicians feel that nephrectomy be performed only on those patients who show response to systemic therapy.[30] The plus points are avoidance of morbidity, mortality, and cost-associated with nephrectomy. Experimental evidence shows that surgery itself can lead to immunosuppression and decreased response to immunotherapy. Platelet-derived growth factor and TGF released during surgery can augment the tumor growth. Some studies have shown that tumor progresses after nephrectomy in 22% of patients.[31] This has been hypothesized to be due to the loss of angiostatin, an angiogenic inhibitor secreted by the primary tumor. This might have been inhibiting the growth of metastases partially.

Other advantages of this approach include earlier initiation of the systemic therapy, the potential for reduction of metastatic and primary tumor burden before surgery, early identification of patients who will benefit from surgical removal of the primary tumor, and the opportunity to examine the effects of systemic therapy on urological tumors.

It is prudent to delay nephrectomy to assess the response to a course of systemic therapy. The most significant benefit of the neoadjuvant approach in the treatment of mRCC is that it can act as a litmus test to select patients who are responding to therapy and most likely to benefit from the proposed cytoreductive nephrectomy. Some tyrosine kinase inhibitors (TKI) even downstage the primary tumor rendering subsequent nephrectomy technically easier.[32] The downside of TMT is that it may increase the surgical morbidity and postoperative complications. This is mainly due to the inhibition of the vascular endothelial growth factor receptors and related pathways. These proangiogenic pathways have important role in tissue integrity. Hence, any disturbance in these could lead to increased incidence of delayed wound healing, fascial disruption, and incisional hernia. This might also cause impairment in the natural regeneration of the microvasculature and predispose the patient to postoperative bleeding and thrombotic events.[33] Tyrosine kinase inhibitors are very costly. A course of 1 month therapy costs about rupees 2 lakhs. This is one of the factors which may militate against this therapy.

Rackley and associates found that patients treated with initial immunotherapy had slightly higher objective response rates and longer median survival rates when compared to patients who had initial nephrectomy and adjunctive immunotherapy.[21] They reported on 62 patients, 37 of whom underwent nephrectomy prior to immunotherapy and 25 patients who received IFN ± IL-2. Three of the 25 patients responded to the immunotherapy and proceeded onto nephrectomy. Of these 3, 2 patients were alive at 18 and 42 months. In this small series, prior nephrectomy group had an 8% response rate and 12 months median survival, whereas, in the initial immunotherapy group, the response rate was 12% and median survival was 14 months.

Krishnamoorthy *et al*. from Cleveland treated 14 patients with mRCC initially with immunotherapy and later 9 patients responding to this with nephrectomy. IL-2 alone or in combination with IFN was given in this study. All patients were then rendered disease free by surgical excision of both residual metastatic disease and primary tumor. Cancerspecific survival at 3 years was 81.5%.[34] Overall, cytokine therapy before nephrectomy did not yield comparable results.

Targeted therapies are dramatically changing the landscape of advanced kidney cancer. Although several studies demonstrated that targeted agents are generally well tolerated, there are limited data on the safety of surgical resection in patients after targeted therapy. Thomas and associates recently reported neoadjuvant targeted therapy followed by nephrectomy.[35] They treated 19 patients with targeted molecules. Ten patients had prior nephrectomy. A median of 4 cycles of sunitinib was given to 12 patients, sorafenib to 3 patients, and bevacizumab to 4 patients. The indication for neoadjuvant-targeted therapy was unresectable primary tumor or the inability to perform nephron sparing surgery in those with bilateral disease. 9, 3, 6, and 3 patients underwent nephrectomy, partial nephrectomy, local recurrence excision, and metastasectomy, respectively. In these two patients with extensive bilateral disease, partial nephrectomy could be done due to the downsizing of the tumor by the targeted therapy. Three patients (16%) had major complications like perioperative hemorrhage, disseminated coagulation, and anastamotic leak. Two patients had minor wound complications. At a median follow-up of 8 months, 16 patients were alive and 8 patients showed disease progression. Margulis and coworkers from the MD Anderson cancer center treated 44 patients with neoadjuvant-targeted molecules.[36] Fifteen patients received sunitinib, 12 patients sorafenib, and 17 patients bevacizumab. Upfront nephrectomy was done in 58 well-matched patients. At analysis around 1 year, 18.2% of patients in the first group and 31% patients in the second group died of RCC. Complications were seen in 32.4%. Withholding targeted therapy for at least two to three half lives before and after surgery may help prevent the adverse effects of these agents on microvasculature and tissue integrity. Half life of temsirolimus is 17 h, sorafenib is 1-2 days, sunitinib is 4 days, and bevacizumab is 17 days. The terminal half life of these agents and their metabolites would suggest that interrupting therapy for 7-10 days before and after surgery would reasonably reduce surgical risks. In addition, the meticulous surgical technique and good hemostasis go a long way in curtailing the side effects.

Most systemic therapy protocols for mRCC were tried before the availability of targeted therapies. They utilized immunotherapy which did not affect the primary tumor. With the recent availability of powerful tyrosine kinase inhibitors which have been shown to reduce the size of the primary tumor, ‘medically selected’ patients (neoadjuvant systemic therapy) for cytoreductive nephrectomy may have better survival. This needs further documentation using randomized controlled studies.

## ROLE OF NEPHRON SPARING SURGERY

The role of nephron sparing surgery (NSS) has been recently examined in the metastatic setting. Besides the preservation of renal function, the additional benefits of NSS include improved performance status, elimination of paraneoplastic syndromes, and eradication of the source of new metastases. Kranbeck *et al*, recently showed that the survival in 14 patients with mRCC who had NSS and 40 patients who had radical nephrectomy was comparable.[37] The sample size was small in this study and selection bias might have crept in. More recently, Hutter and colleagues have shown in a well-matched study that NSS did not undermine the RCC-specific survival.[38] This study included 38 patients having NSS and 99 patients who had radical nephrectomy. The median actuarial survival of the NSS vs radical nephrectomy patients was 5.1 vs 3.3 years. Krishnamoorthy *et al*, from Cleveland, reviewed the outcome in 15 patients with mRCC who had NSS and surgical or systemic treatment of metastases. All cases were technically successful and the need for renal replacement was found in only one patient.[39] These studies showed a survival advantage for the NSS group. This may partly be due to the preservation of the renal function. Recent studies have shown that chronic kidney disease (CKD) is present in 26% of apparently normal patients with small renal tumors and normal serum creatinine. Casual nephrectomy can lead to worsening of the renal function. Patients who had nephrectomy had a reduced survival due to increased mortality from cardiovascular causes The likelihood of developing chronic kidney disease with a GFR of <45 ml/min/1.73 m^2^ after partial nephrectomy is <5%, whereas after radical nephrectomy it is 36%.[40]

## THE ROLE OF METASTASECTOMY IN MRCC

Patients with mRCC usually have a dismal prognosis. However, with the introduction of TMT the outlook has dramatically changed. Favorable subgroups include solitary metastases and DFI to metastases of >1 year. Complete resection of isolated metastases was associated with 5 year survival rates of between 35 and 60%. Findings from Mayo clinic, Memorial Sloan Kettering Cancer Center (MSKCC), and from Martin Luther university showed a 5-year survival of 30-50% following metastasectomy. Interestingly, even when the likelihood of complete resection was low, metastasectomy still maintained its beneficial effect.[41]

### Prognostic variables

Features adversely associated with survival in patients with mRCC of clear cell variety include constitutional symptoms at nephrectomy, metastases to bones or liver, multiple metastases, metastases at nephrectomy or within 2 years of nephrectomy, tumor thrombus level 1-4, nuclear grade 4, and coagulative necrosis. Complete resection of all metastases was associated with improved survival.

Patients with liver metastases are more likely to die of RCC compared to metastases to bone (69% vs 35%). A study by Han *et al*, showed that the number of metastases rather than the site of metastases is more important regarding survival.[42]

Complete resection of metastases was associated with a twofold decreased risk of death from RCC. A recent algorithm by Motzer *et al*, has suggested that the Karnofsky’s performance status (KPS), lactate dehydrogenase (LDH) level, hemoglobin(Hb) level, serum creatinine, and time from the diagnosis to immunotherapy were significantly associated with survival.[43]

In a study of 670 patients with mRCC treated at MSKCC, the following factors were considered to be associated with poor survival: low KPS (<80%), high LDH (>1.5 × upper limit of normal), low Hb(lower than the lower limit of normal), high corrected serum calcium (>10 mg/dl), and absence of nephrectomy.[43] Median survival ranged from 4 to 13 months. For patients without any of the above risk factors the median time to death was 22.1 months. For patients with only one of the factors the time to death was 12 months and with multiple factors, only 5 months.

### Role of lymphadenectomy

Parker has demonstrated that the primary regional drainage of kidney is predicatable.[44] On the right side, the regional nodal drainage is to the lateral caval, pre-caval, post-caval, and inter-aortocaval nodes. On the left side, para-aortic, pre-, post-, and inter-aortocaval nodes. However, the secondary drainage is variable and unpredicatable. Despite the predicatable nature of the renal lymphatic drainage, patterns of lymph node metastasis in patients undergoing surgery for RCC are rarely predicatble. Because the role of lymph node dissection in mRCC is less well studied than in localized and node only disease, the decision for lymphadenectomy (LND) is heavily influenced by the experience and the bias of the surgeon. For patients with lymph node (LN) metastases, the median survival was 5 months longer for patients who had LND than for patients who did not. This difference was statistically significant.

While the primary intent of LND in localized RCC is to increase the detection of nodal micrometastases thereby improving staging accuracy, in N + M0 disease, the principal goal is complete disease resection and cure. However, only a small group of patients have lymph node only disease (4-10%). Giuliuni and coworkers reported a 5 year and 10 years survival of 47.9% and 31.9%, respectively, for N + M0 disease following an extended LND.[45] In another study by Peter and colleagues, the 1 and 5 years survival following extended LND was 87.5% and 43.75%, respectively. Without LND, the rate declined to 56.5% and 25.69%, respectively.[46]

In advanced disease, the presence of lymph nodes with wide spread metastases portends a grave scenario. In a study by Peter and Brown, patients with mRCC were subjected to nephrectomy + lymphadenectomy, nephrectomy, and no surgery.[46] The survival at 1 and 5 years for the three groups were as follows: 81%, 47.7%, and 32.3% for the first category, 28.9%, 9.1%, and 11.4%, respectively. An UCLA study also reported similar benefits.[47] Recently, Patard and coworkers reported complete LND after sunitinib in a patient who was initially found to have unresectable lymph nodes. There was no recurrence at 6 months.[48] In patients undergoing LND, the most frequent complication was bleeding (>1 L) which occurred in 10% of patients. Other complications included pleural damage, infection, and lymph leak. The overall complication rate was 25.7%. Because, nodal positive disease represents an aggressive phenotype of RCC, these patients are in desperate need of an effective adjuvant therapy.

Accumulated evidence is in favor of adding LND in lymph node only disease (which is rare) or when there are limited and resectable metastases elsewhere.

### Lung metastases

Lungs are the most common sites of metastases in RCC patients. Resection of pulmonary metastases (LM) is associated with higher survival rates and results are better when compared to other anatomical sites. Factors generally agreed upon to impart longer survival postoperatively are fewer pulmonary metastases, lack of lymph node involvement, pathological evidence of complete resection, and the synchronous or metachronous nature. The number of resected lung metastases has little influence on survival when the resection is complete. Surgery for lung metastases related to primary RCC is safe and curative in one-third of patients. The most important predictive factor for a longtime survival is the completeness of resection. The first resection of a pulmonary metastasis in a patient with RCC was performed by Barney and Churchill in 1939.[49] Since then surgery remained the only effective treatment for patients with isolated lung metastases. The published 5-year survival rates after metastasectomy of renal origin range from 36 to 54%. Patients who developed metachronous metastases had far superior survival rates than those who had synchronous metastases (5-year survival of 56.7 months vs 15.3 months). Hoffman *et al*, suggested that pulmonary metastases resection can be done if the DIF was long and the number of metastases upto 6 and good functional status.[50] Repeat metastasectomy for recurrent pulmonary metastases appears to be efficacious in certain patients since the group from the Mayo Clinic reported that the 5-year overall survival in this subgroup was similar to that in patients without recurrence.[51] Zagoria and colleagues treated lung metastases with radiofrequncy ablation.[52] At 1-year follow-up there was no recurrence. Thoracoscopic techniques are being routinely used nowadays for resection of the metastases. Soga and associates treated 39 patients with unresectable pulmonary metastases with RFA. The recurrence free survival rates were 92% at 1 year, 23% at 2 years, and 23% at 5 years.[53] Radiofrequency ablation is considered safe and effective treatment modality for prolonging survival in patients with unresectable secondaries. As the 5-year survival after metastasectomy of lung nodules is 36-54%, resection of the secondaries should be considered whenever feasible.

Pulmonary metastases are shown to be significantly more susceptible to IFN treatment compared to other sites. However, no activity of IFN has been found in this adjunct setting and treatment has often resulted in increased morbidity. Therefore, adjunct IFN therapy cannot be routinely recommended after treatment of pulmonary metastases.[54]

### Adrenal metastases

The incidence of ipsilateral adrenal metastases from RCC varies from 1.1 to 10%.[55] The higher incidence of metastases are seen in patients with upper pole tumor, left sided tumors, and larger tumors. Adrenals can be involved in different ways viz: directly via the Gerota’s fascia, through the vessels piercing the Gerotas fascia, lymphatics, and directly as arterial emboli and retrograde venous embolization.

Many cases of ipsilateral and contralateral adrenal metastases were reported even with small lower pole tumors.[56] Therefore, the sensitivity, specificity, and predictive value of the predisposing factors are insufficient to define all the cases at risk. CT scan has been shown to be highly sensitive for the diagnosis of adrenal metastases. If a preoperative CT scan shows normal adrenal, then the chance of adrenal metastases is very low and probably does not need to be resected except in large upper pole tumors.[57]

In patients without any systemic spread and intra-adrenal metastases only, the median long term survival was 11.7 years. Patients with adrenal metastases and systemic spread had a survival of 16 months. Patients with RCC and a single contralateral adrenal metastasis should be considered as having solitary metastases. These patients are recommended radical nephrectomy, ipsilateral adrenal exploration, and contralateral adrenalectomy as long as there are no other metastases. The ipsilateral adrenal should only be removed if there is any suspicion of metastasis.[58] Patients with synchronous contralateral adrenal metastasis are suitable for ipsilateral adrenal preservation. Right-sided lesions are more amenable for preservation as opposed to left in view of lower frequency of metastasis and different anatomical relationship. Patients with bilateral synchronous adrenal metastases should be considered to have disseminated disease. Yu *et al*, in a recent report showed that patients who had bilateral adrenalectomy, on an average died within 6 months.[59] Radical nephrectomy in these patients is only palliative. Paul *et al*, reported a new algorithm to determine the risk of adrenal metastasis.[60] Adrenalectomy was considered unnecessary if the maximum diameter of the tumor was <8 cm, and staging examinations did not show organ or lymph node metastases. The clinical value of adrenalectomy is as low as LND. Recent studies indicate adrenal involvement to be a poor prognostic indicator.[61]

### Metastases to bones

Metastases to bones from RCC is common (30-40%).[62] These are usually highly vascular and destructive lesions. They pose unique surgical challenges due to the risk of life-threatening bleeding and resistance to other forms of treatment. Osseous metastases in RCC bring about with them poor performance status due to the intractable pain and pathological fracture. The surgical procedures done in these patients included curettage and cementing, internal fixation, en bloc resection, amputation, and nailing. Only patients with good performance status and solitary metastases usually underwent surgery. Surgery for patients with spinal and pelvic metastases is usually associated with higher morbidity when compared with long bone metastases which can be done with a minimally invasive method.[63] Toyada *et al*, have proposed two prognostic factors in the treatment of bone metastases with RCC and accordingly categorized these patients into good prognosis group and bad prognosis group.[63] The two factors are the time interval from nephrectomy to the appearance of the bone metastases and the presence of extra-osseous metastases. If the metastases developed within 2 years, the prognosis was bad. In their 50 patients, they found that those with poor prognostic factors had a median survival of 5 months while those in the good category had 30 months’ median survival.

Yuvraj *et al*, found that the number of metastases and the synchronous or metachronous nature of the metastases also are important.[64] In their study of patients with mRCC and solitaty metastases to the bone, 6 patients had synchronous and 13 patients had metachronous metastases to the bones. The former had a median disease free survival of 25 months and the later group had 63 months.

Hence, it appears that the most important prognostic factors in these patients are the number of metastases, time from nephrectomy, and presence or absence of extraosseous metastases. The data available from published reports indicate that in patients with limited disease, with the presence of a solitary metastasis, with present or impending pathological fracture or intractable pain and neurological symptoms, surgical treatment not only gives effective relief but also significantly improves prognosis in a selected group of patients.

### Liver metastases

Involvement of the liver occurs either by contiguous extension or hematogenus spread. A large renal tumor may indent or compress the liver but actual invasion is rare. Hematogenous spread is more common. When intrahepatic metastases are present, 98% of patients have other metastases as well. Most of these patients are symptomatic, however, the liver function in most are normal. Partial hepatectomy in direct extension gives a good survival. Complete resection of metachronous liver metastases can be achieved in the majority of patients. However, significant morbidity and mortality as well as the limited prognosis even after R0 resection strongly suggest careful patient selection. The prognosis of patients with either metastatic disease stage IVb or contiguous spread of renal cell carcinoma to adjacent organs (stage IVa) has been uniformly poor, with a 1% 3-year survival rate and less than a 5% 5-year survival rate, respectively.[7] Two recent reports of RCC metastases to liver had a complication of 31-36%. The 2-year survival was 56%. Small sized metastases, evidence of complete resection, and shorter DFI were favorable in terms of survival.[65]

Patients with hepatic secondaries are considered for other modalities of ablative therapy only if they are not candidates for surgical resection, have <4 metastases, and lesions are <5 cm. Most studies are retrospective, short-term reports and no definite conclusions regarding survival can be made.[66] Radiofrequency ablation has been the most studied in hepatic secondaries. However, radiofreqency ablation provides effective palliation for these patients.

### Metastases to brain

The largest recent series of patients with RCC undergoing surgical resection of brain metastases was presented by the group from MSKCC. In this study the mean survival after metastasectomy was 12 months.[67] Notably longer DFI was not associated with improved survival. Recently, Vecil and Lang reviewed the M D Anderson Cancer Center experience with a specific subset of patients, that is those with intraventricular brain metastases who underwent metastasectomy.[68] Of the 35 patients, 16 had RCC, which is more than any other histology that was identified. The operative complication rate was12% and there was no perioperative mortality. The median survival in patients with a single metastasis was 13.6 months. Bevacizumab is contraindicate in patients with brain metastases because of its tendency to cause increased bleeding. Not uncommonly, patients with mRCC present with metastases exclusively in the choroid plexus. Most of these patients present with intraventricular bleed and consequent neurological deficits. CT and MRI scans are useful in diagnosing this condition. Almost all cases reported were solitary lesions. Resection of the lesion, steriotactic surgery, and chemotherapy have produced good results, and survival up to 5 years have been reported.[69]

### Metastases to thyroid

Thyroid is a highly vascular organ yet the incidence of metastases in the thyroid is rare. Thyroid receives approximately half the volume of arterial blood received by the entire liver. RCC is the most common source of secondaries in the thyroid (56%). The probable reasons for the rarity of metastases in the thyroid are initial filtration of the malignant cells by the lungs; even if they reach the thyroid, due to the high volume of high velocity blood flow, the tumor cell may not be able to get a foothold there; high concentration of oxygen and iodine inhibits the proliferation of fixed tumor cells. If a patient with a history of nephrectomy for RCC subsequently has a solitary thyroid mass, one should consider isolated thyroid metastasis as well as a primary thyroid tumor. After thyroidectomy or lobectomy for metastases from RCC, 60% were disease free at 6 years. In a report by Isalymph nodeieks and associates, the overall 5 year survival rate in 45 patients following thyroid metastasectomy was 51%.[70] Nineteen patients died during the study. According to these authors, the overall survival of patients undergoing thyroidectomy for metastases from RCC is affected rather by the general health of the patient than the tumor-related factors. There is a significant coincidence of thyroid and pancreatic metastases of RCC.

### Pancreatic metastases

Pancreas is a rare target for metastases from RCC. About 50% of the metastases to the pancreas are asymptomatic. Metastases move to the pancreas via lymphatics and the venous channels. Nagakawa has shown lymphatics from the head of the pancreas to the dorsal aspect of the renal artery.[71] Lore *et al*, proposed that diseases of the pancreas can lead to alterations in the portal blood flow and opening up of the renal portals from the pancreas.[72] In the absence of other metastases and solitary metastases to the pancreas, the 5 year survival is about 31%.[73] Mortize *et al*, reported 10 month survival for patients after various pancreatic surgeries for patients with metastases from RCC.[74] LND is usually not required as no cases of lymph node metastases have been reported in this setting. The usual surgeries done in these patients include classical Whipple’s, total pancreatectomy, and distal pancreatectomy. Short-term survival (upto 1 year) has been reported after ablation of the pancreatic secondaries using radiofrequency energy.[75]

### Metastases to other organs

Renal cell carcinoma can metastasize to almost any organ in the body even as late as 20 years. Oligometastases and long interval from radical nephrectomy are favorable indicators of survival following metastasectomy.

## CONCLUSION

Patients with mRCC are mostly symptomatic and badly in need of effective palliative therapy. As the natural course of the disease is highly variable, therapy needs to be individualized. The management of these patients has undergone dramatic changes mostly due to the introduction of TMT. The natural history of the disease can be changed by a range of these agents. The era of TMT has only begun. Many more drugs are in the pipeline and these may have a greater impact on the survival.

A multimodal approach is the current standard of treatment for patients with mRCC. Selected patients with oligometastatic diseases, long period of interval from radical nephrectomy to the development of metastases, and good performance status are the most important factors that have an impact on the survival of these patients. Even though cure is not possible, improved survival and quality of life can be achieved with combination therapy using cytoreductive nephrectomy and systemic therapy. Laparoscopic resections are being increasingly done to reduce the morbidity. Alternative ablative methods such as radiofrequency ablation, cryosurgery, etc may play a greater role in these patients in the future. Currently, upfront cytoreductive nephrectomy followed by systemic therapy is the standard. However, in the days to come, planned and well-controlled clinical trials using TMT in the neoadjuvant setting may completely change this, hopefully, providing better survival and quality of life.
